# National survey of older people’s community mental health teams in England

**DOI:** 10.1192/bjo.2025.10958

**Published:** 2026-01-26

**Authors:** Malvika Muralidhar, Hannah Chapman, Oliver Kelsey, Jasmine Shaw, Grace Shepherd, Felicity Pearce, Charlotte Kenten, Harriet Demnitz-King, Elizabeth L. Sampson, Greta Rait, Ruth Dobson, Joanna Brown, Yvonne Birks, Naaheed Mukadam, Christoforos Pavlakis, Marie Fitzgerald, Sube Banerjee, Claudia Cooper

**Affiliations:** Wolfson Institute of Population Health, Queen Mary University of London, London, UK; North East London NHS Foundation Trust, London, UK; Primary Care and Population Health, University College London, London, UK; School for Business and Society, University of York, York, UK; Division of Psychiatry, University College London, London, UK; PPI Contributor, UK; Faculty of Medicine and Health Sciences, University of Nottingham, Nottingham, UK

**Keywords:** Dementias/neurodegenerative diseases, community mental health teams, patients and service users, prevention, survey statistics (or survey methods)

## Abstract

**Background:**

The mental healthcare workforce supporting people with dementia and comorbid mental disorders requires specific skills and knowledge.

**Aims:**

We co-designed and conducted a survey to understand key issues facing community mental healthcare services accessed by older adults.

**Method:**

We invited all English National Health Service (NHS) older people’s community mental health teams (OPCMHTs) in England to complete the survey. We compared service structures, resourcing and waiting times between regions, and considered how responses might inform current policy priorities.

**Results:**

A total of 182 out of 242 (75.2%) English NHS OPCMHTs participated. We estimated there were 120 233 referrals to OPCMHT services per year, with 77.5% of services reporting increasing referral rates. In a quarter of services (*n* = 46, 25.3%), clients waited over a month from referral to initial assessment. Most services (107/181, 59.1%) experienced difficulties accessing in-patient beds for people with dementia, with rural regions more likely to report these difficulties. Half of the services (*n* = 100, 55.2%) reported providing higher-quality care for people with dementia than 5 years ago, despite increasing caseload complexity. Resource limitations challenged opportunities for prevention, care quality and collaborative working, and respondents rated team relationships with social services (*n* = 86, 47.8%), general hospital in-patient (*n* = 74, 41.4%) and out-patient (*n* = 54, 30.2%) services, and primary care (*n* = 54, 30.2%) as poor or requiring improvement.

**Conclusions:**

OPCMHT service leads are committed to integrated working, but services are insufficiently resourced to realise their potential. Addressing challenges related to workforce retention, training and ways of working could optimise OPCMHT contributions to integrated care for people with dementia.

An estimated 982 000 people have dementia in the UK, and this will rise to 1.4 million by 2040.^1^ People with dementia experience mental illness more often than the general older population,^2,3^ for several reasons. Depression is a prodromal feature of,^4^ and risk factor for, dementia.^5^ Older adults with schizophrenia are also at increased dementia risk.^6^ Three-quarters of people with dementia experience at least one clinically significant behavioural or psychological symptom, including depression, anxiety, behaviour that challenges and psychotic symptoms.^7^ These symptoms reduce quality of life^8^ for individuals and their carers, and increase formal care costs.^9^

Supporting people with dementia and comorbid mental disorders is a major global challenge. Studies in the USA have noted greater usage of emergency departments by people with dementia, and suggest that this may relate to mental disorders.^10^ The international literature notes the importance of providing specific skills and knowledge to the dementia health and care workforce.^11–13^

In England, the National Audit of Dementia highlighted considerable variation in both how dementia is diagnosed and in early post-diagnostic care.^14^ Our national survey of English memory services also revealed significant variation and inequalities between regions and service types.^15^ Between 2025 and 2027, national dementia audits are planned in general acute hospitals and dementia diagnostic services, but not community mental health services responsible for treating mental comorbidities in people with dementia.^16^ In England, community specialist mental health provision for people with dementia is provided by National Health Service (NHS) community mental health teams (CMHTs). There has been considerable flux in how older people’s community mental health teams (OPCMHTs) are organised over the past decade. Many localities operate distinct memory services to diagnose and provide post-diagnostic dementia support, whereas others combine this function with OPCMHTs or CMHTs for adults of all ages.^17^

Appropriate treatment of comorbidities in people with dementia must consider interactions between cognitive, physical and mental disorders, including risks of delirium, and the wider relational and social context of living with dementia.^18^ High-quality services can markedly reduce morbidity^19^ and mortality.^20^ A survey of 429 OPCMHTs in England in 2008–2009, found that less than a third contained the full complement of professionals recommended by government policy, or offered the range of liaison activities anticipated in the National Dementia Strategy.^21^ A 2012 survey echoed these findings, with only 18% of OPCMHTs having staff with allocated time in care homes.^22^

The current survey is the first of English OPCMHTs for over a decade. We aimed to explore how current provision maps to policy priorities. In July 2025, the UK Government launched the 10-Year Health Plan for England,^23^ building on the Darzi report into the state of the NHS.^24^ The Plan prioritises three shifts: from hospital-based to community care; from analogue to digital; and from sickness to prevention-focused, integrated care. Neighbourhood health services could significantly benefit people with dementia, most of whom live with multiple comorbidities.^25^ Plans to develop modern service frameworks cited early priorities as cardiovascular disease, mental health, and frailty and dementia. In 2021, NHS England proposed that adults and older adults presenting to community-based mental health services should start to receive help within 4 weeks of referral,^26^ but OPCMHT services are operating in challenging contexts, and demand and waiting lists for community services have surged.^24^

## Objectives

This is part of a broader set of surveys of social care, primary, and secondary health services in England, to be conducted between 2024 and 2026.^15^ Our objectives were:

- to describe service structures, and procedures of community mental health services for older people, including people with dementia in England;
- to explore whether there are inequalities between regions in levels of resource (approximated as the number of referrals per full-time equivalent (FTE) staff member) and waiting times for community and inpatient services, and whether clients are waiting less time in regions with more resourced community teams;
- to explore what changes in the past 5 years make service leaders feel proud and cause them concern, what changes they anticipate in the next 5 years and how these might align with the three ‘big shifts’ in current government health policy in England.

## Method

The survey was conducted within the NIHR Dementia and Neurodegeneration Policy Research Unit at Queen Mary University of London (DeNPRU-QM), commissioned by the English Department of Health and Social Care and approved by the National Research Ethics Service and Health Research Authority on 14 May 2024 (approval number 24/IEC08/0008).

### Survey design

We consulted patient and public involvement (PPI) members and obtained their feedback on an initial draft of the survey. We then purposively invited people from academic, clinical and policy backgrounds across regions in England and staff roles, to three video call, co-design workshops in October 2024. Previous research on OPCMHT service structure and processes,^21,22,27^ and policy documents,^28,29^ circulated before the workshops, guided survey development. Using a structure, process and outcome framework, group members proposed and discussed potential survey items to support our aim to explore how current provision maps onto government policy priorities. All attendees were sent the final survey and invited to provide further comments.

The survey comprised questions on service characteristics, including structure (e.g. older adult pathways, staffing mix, referral numbers), and processes (e.g. wait times for assessment and subsequent services, availability of evidence-based interventions and support). It also included questions about joint working, liaison and support activities, service changes over the past 5 years and anticipated changes in the next 5 years. The survey was specifically developed for this study (provided in Supplementary File 4 available at https://doi.org/10.1192/bjo.2025.10958).

### Survey sample and data collection

A sampling frame of all OPCMHT services in England was developed from publicly available information. We identified services across Mental Health Trusts under each Integrated Care Board (NHS organisations responsible for planning health services for their local population), in an iterative process to account for mergers, and integration of older adult mental health services (i.e. services that combined working adult and older adult teams), resulting in a final sampling frame of 242 OPCMHT services, who were invited to participate between November 2024 and June 2025.

Responding services were asked to identify a staff member with knowledge of service structures, processes, outcomes, historical development and future trends. After providing informed consent, the staff member completed the survey with a researcher by video call, or self-administered if they preferred. All surveys were conducted via the online survey platform Qualtrics (https://www.qualtrics.com). Participants did not receive compensation.

Researchers obtained publicly available area-level data regarding rurality and quality of services.

The Local Authority District rurality was obtained using the 2021 Office for National Statistics classifications;^30^ 1 (majority rural: majority further from a major town or city), 2 (majority rural: majority nearer to a major town or city), 3 (intermediate rural: majority further from a major town or city), 4 (intermediate rural: majority nearer to a major town or city), 5 (intermediate urban: majority further to a major town or city), 6 (intermediate urban: majority nearer to a major town or city), 7 (urban: majority further to a major town or city) and 8 (urban: majority nearer to a major town or city).

This was recategorised as ‘rural’ (rural or intermediate rural, categories 1–4) or ‘urban’ (intermediate urban and urban, categories 5–8).

Service quality was obtained from the Care Quality Commission ((CQC) regulator of all health and social care services in England) rating of older people’s services within each Trust.^31^

### Data analysis

Data were exported from Qualtrics and analysed using Statistical Package for the Social Sciences (SPSS; version 29 for Windows; IBM, Armonk, New York, USA; https://www.ibm.com/products/spss-statistics). We summarised findings descriptively. For each NHS region (standard grouping of regional teams who support local systems), we estimated total number of FTE staff in OPCMHTs, assuming the median value for non-responding services.

We asked about the number of annual referrals for each service, and estimated the number per FTE, applying the median estimated number of referrals and FTE per service in each region for non-responding services. Where services could not estimate the number of referrals, we treated this as missing data. We applied this rule to four services who provided number of referrals to both memory services and OPCMHT, or a service wide number of referrals to a trust single point of referral. Where services provided the number of accepted referrals (*n* = 3), we assumed this was a reasonable estimate of referrals received. We compared, without formal statistical testing, how the resources per referral in each region compared with the waiting times for the service.

We conducted content analysis of responses to open-ended questions, using established methods.^32^ We used UK Government policy as a framework, exploring how challenges and opportunities aligned with three proposed ‘big shifts’: from hospital to community; analogue to digital; and sickness to prevention^23^ to develop our coding framework, combining deductive and inductive analytic approaches.^33^ M.M. and C.C. independently coded >10% of responses for each question, developed initial groupings of themes and then met to discuss a coding framework. Two authors (M.M. and O.K.) then coded the remaining responses.

## Results

### Survey response

A total of 159 staff participated, representing 182/242 (75.2%) of the teams approached. Transitions in services meant that two services were surveyed twice, with only the most recent responses included. Respondents included 76 team managers/leaders, 30 operational area, sector or service managers, 23 senior nurses/advanced or clinical practitioners, 13 clinical leads, ten psychiatrists, three service heads/directors, one clinical psychologist, one neurologist, one manager and one performance resource lead. Eleven respondents with pan-service operational roles or who worked across more than one team completed surveys for multiple (≤7) services. Participating staff members had been employed in their current role for a mean of 4.9 years.

Table 1 shows the distribution of participating services by region. Completion rates were highest in the London NHS region (28/30, 93.3%) and lowest in the North-West region (13/24, 54.2%).

**Table 1:** Proportion of services who completed the survey from each NHS region

NHS region	Services participated, *n*	Proportion, %
East of England	24/26	92.3
London	28/30	93.3
Midlands	29/44	65.9
North-East and Yorkshire	36/46	78.3
North-West	13/24	54.2
South-East	35/44	79.5
South-West	18/28	64.3

NHS, National Health Service.

### Service structure

Most (128, 70.3%) of services were in urban or intermediate urban areas; and rated as good (152, 83.5%) at most recent CQC inspections (Table 2). A total of 155/182 (85.2%) services worked alongside a distinct memory service for assessment and diagnosis of dementia, although 133 of these (85.8%) also sometimes diagnosed dementia. In the remaining 27 (14.8%) services, memory and community mental health services were combined, with no separate memory service. One hundred nineteen (76.8%) of these services were exclusive to older adults aged above 65 years, and 36 (23.2%) included adults of all ages (i.e. working age and older adults).

**Table 2:** Summary of service characteristics of services

Characteristic	Finding
Rurality (*n* = 182), *n* (%)	
Rural	54 (29.7)
Urban	128 (70.3)
CQC rating for older people’s services within Trust (*n* = 182), *n* (%)	
Outstanding	24 (13.2)
Good	152 (83.5)
Requires improvement	6 (3.3)
Annual referral rate (*n* = 160)^a^, median (IQR)	
Past year	500 (286–850)
Per FTE staff member	31.5 (18.7–46.3)
Change in referrals relative to 5 years ago (*n* = 182), *n* (%)	
Increased	141 (77.5)
Decreased	9 (5.0)
Stayed the same	32 (17.6)
Median (IQR) proportion of FTE clinical staff who are: (*n* = 167)^a^ (%)	
Mental health nurses	40 (33–50)
Support workers/Band 4 therapists	14 (10–21)
Psychiatrists	11 (8–15)
Occupational therapists	9 (6–12)
Clinical psychologists	6 (4–9)
Social workers	0 (0–5)
Consultant nurses/advanced practitioners	0 (0–6)
Provision of home treatment/crisis services (*n* = 182), *n* (%)	
Yes	46 (25.3)
Separate team	128 (70.3)
No such team exists	8 (4.4)
Provision of care home liaison (*n* = 182), *n* (%)	
Yes	88 (48.4)
Separate team	58 (31.9)
No such team exists	36 (19.8)
Place of initial assessment (*n* = 179), median (%)	
At home	80 (60–95)
In the clinic	15 (5–30)
Online/telephone	0 (0–1)
Other	0 (0–0)
Average waiting time to receive an initial assessment (*n* = 181), *n* (%)	
1 week	22 (12.2)
2 weeks	49 (27.1)
1 month	59 (32.5)
Over 1 month	46 (25.4)
Not sure	5 (2.8)
Wait time (in weeks) for services after initial assessment, median (IQR)	
Clinical psychologist	8.0 (4.0–12.0)
Social services assessment	4.0 (2.6–7.0)
Medical out-patient review	4.0 (2.5–7.0)
Speech and language therapist	4.0 (1.1–9.5)
Physiotherapy assessment	3.0 (1.8–6.0)
Occupational therapy assessment	3.0 (2.0–5.0)
Dietician	2.0 (2.0–3.0)
Allocation to a care coordinator	1.0 (0.0–2.0)
Change in proportion of clients presenting with: *n* (%)	
Dementia (*n* = 179)	
Increased	139 (77.7)
Decreased	1 (0.6)
No change	39 (21.8)
Harmful use of alcohol (*n* = 178)	
Increased	118 (66.3)
Decreased	4 (2.3)
No change	56 (31.5)
Harmful use of illicit drugs (*n* = 177)	
Increased	46 (26)
Decreased	3 (1.7)
No change	128 (72.3)
Personality disorder (*n* = 176)	
Increased	111 (63.1)
Decreased	4 (2.27)
No change	61 (34.7)
Late-onset psychosis (*n* = 174)	
Increased	52 (29.9)
Decreased	5 (2.87)
No change	117 (67.2)
Anxiety (*n* = 176)	
Increased	123 (69.9)
Decreased	3 (1.7)
No change	50 (28.4)
Depression (*n* = 177)	
Increased	119 (67.2)
Decreased	1 (0.6)
No change	57 (32.2)
Autism (*n* = 176)	
Increased	73 (41.5)
Decreased	1 (0.6)
No change	103 (58.5)
Cognitive impairment following pre-existing psychosis diagnosis (*n* = 175)	
Increased	85 (48.6)
Decreased	2 (1.14)
No change	88 (50.3)
Ongoing backlog since COVID-19 pandemic (*n* = 181), *n* (%)	
Yes	38 (21)
No	141 (77.9)
Not sure	2 (1.1)

CQC, Care Quality Commission, IQR, interquartile range; FTE, full-time equivalent.

a. *n* refers to number of services who provided a response to the question.

Overall, most services (145/182, 79.7%) had distinct services for older adults (typically defined by frailty and need), of which 26 combined memory service and community mental health functions. Thirty-seven (20.3%) services surveyed were responsible for community mental health provision for adults of all ages (of which only one combined memory service and community mental health functions).

A quarter of services (46, 25.3%) provided a home treatment or crisis resolution service, and 88 (48.4%) provided care home liaison services. Eight (4.4%) of the teams had no home treatment/crisis team access and 36 (19.8%) no care home liaison team, whereas in remaining areas, these functions were served by separate teams.

### Staffing

Most staff employed were mental health nurses (median 40%, interquartile range 33–50%), support workers or Band 4 therapists (median 14%, interquartile range 10–21%), or psychiatrists (median 11%, interquartile range 8–15%). Seventy-three (40.1%) services were fully staffed. Others (109/182, 59.9%) had unfilled posts, most frequently for nursing, medical consultant and clinical psychology positions (Table 2).

### Referrals

Services had received, on average a median of 500 (interquartile range 286–850) referrals in the past year, or 31.5 per FTE staff member (Table 2). From this data, we estimated that there were 120 233 referrals to CMHT services for older people in England in the past year, assuming median referral rates applied to non-responding services.

Over three-quarters of services (141/182, 77.5%) reported an increase in referral rates in the past 5 years; and most an increase in older adults presenting with dementia (140/182, 76.9%), anxiety (123/182, 67.6%), depression (118/182, 64.8%), harmful use of alcohol (118/182, 64.8%) and personality disorders (111/182, 61%).

A third of the services (61/161, 37.9%) accepted self-referrals, in three cases only for returning patients. Among the services accepting self-referrals that were able to provide an estimate (*n* = 53), they accounted for 5% (interquartile range 1–7.5%) of referrals in the past year. Almost all other referrals came from primary care.

### Service provision

All services offered home visits to clients with dementia, and most clients (median 80%, interquartile range 60–95%) were seen at home (including in care homes), with others (median 15%, interquartile range 5–30%) primarily seen in clinic (Table 2).

To support people with dementia with comorbid depression and/or anxiety, most services offered individual psychological therapy sessions (142/182, 78.5%), which included trauma-informed therapies, acceptance and commitment therapy, and compassion-focused therapy. Many services also offered individual sessions for family carers (114/182, 63%). Just under half offered groups for people with dementia (88/182, 48.6%), typically cognitive stimulation therapy. Six services (3.3%) reported that none of these therapies were offered within the OPCMHT, and that these were provided by memory services, post-diagnostic support teams as part of dementia pathways, or by referral to external providers. Some services offered specific support for people from minority ethnic groups (34, 18.8%), LGBTQIA+ populations (14, 7.8%) and veterans (38, 21.1%).

Most services (145/182, 80.1%) reported that patients were offered opportunities to take part in research. Overall, more than half of the services (100/182, 55.2%) reported providing higher quality of care to people with dementia over the past 5 years, whereas 29 (16%) felt it was lower quality, 40 (22.1%) that there was no change and 12 (6.6%) were unsure.

### Waiting times

A quarter of services (46/182, 25.3%) reported an average waiting time of over a month for community assessments, from referral to initial assessment (Table 2). Wait times for services after initial assessment were longest for clinical psychologists (median 8.0 weeks, interquartile range 4.0–12.0 weeks). Over a third of services (65/182, 35.7%) allocated care coordinators immediately or had no wait time for allocation as care coordinators carried out initial assessments. Over half (107/181, 59.1%) of services reported difficulty accessing in-patient beds for people with dementia and comorbid mental health problems, and one service did not provide a response. Most services (164/181, 90.6%) believed that service barriers, most commonly delays with care packages and care home allocation, prevented timely discharge into the community.

The proportion of people waiting a month or more for a community assessment ranged from 7.7 to 41.7% (median 26.5%, interquartile range 10.7–29.2%) between regions (Table 3); but with no clear relationship to resourcing or rurality. Teams in the two most rural regions (South-West and East of England) most frequently reported difficulties accessing in-patient beds (88.9% and 75% of services, respectively).

**Table 3:** Referral rates, staffing and indicators of challenges accessing community and in-patient services by region

NHS region	Rurality, *n* (%) rural in each region	Median (IQR) referral/FTE rate	Total FTE (clinical staff) in region services	*n* (%) of services with initial wait time over 1 month	*n* (%) of services reporting difficulty accessing in-patient beds
South-East	8/34 (23.5)	39.0 (25.9–57.1)	937.52	9/34 (26.5)	26/34 (76.5)
Midlands	7/29 (24.1)	35.0 (21–65.8)	609.4	8/29 (27.6)	10/29 (34.5)
North-East and Yorkshire	13/36 (36.1)	31.2 (20.5–35.2)	858.2	15/36 (41.7)	15/36 (41.7)
South-West	14/18 (77.8)	30.2 (19.1–48.6)	424	3/18 (16.7)	16/18 (88.9)
London	0/28 (0)	27.5 (19.7–52.6)	482.8	3/28 (10.7)	14/28 (50)
East of England	10/24 (41.7)	26.0 (14–46.2)	544	7/24 (29.2)	18/24 (75)
North-West	2/13 (15.4)	19.0 (8.1–47.1)	493.2	1/13 (7.7)	8/13 (61.5)

NHS, National Health Service, IQR, interquartile range; FTE, full-time equivalent.

### Interfaces with other services

We asked respondents about regular liaison activities with other services aside from co-working for specific clients: 96/182 (53%) services regularly liaised with primary care, largely through multidisciplinary team (MDT) meetings, or to discuss and update on practices and services (Table 4). A third of services (62/182, 34.3%) had regular contact with care homes, through MDT meetings, training or service improvement. Forty-six (25.4%) services liaised with general hospitals daily to quarterly, whereas over a third (62/182, 34.1%) had regular liaison with other services, including the voluntary sector, neighbourhood mental health teams (services for under 65s who are not clinically frail but present with severe mental illnesses), social care and frailty teams.

**Table 4:** Rating of interface between community mental health teams and other services

Service	Excellent, *n* (%)	Good, *n* (%)	Requires improving, *n* (%)	Poor, *n* (%)	Not applicable, *n* (%)
Memory services (*n* = 181)^a^	78 (43.1)	72 (39.8)	5 (2.8)	0 (0)	26 (14.4)
Mental health wards (*n* = 180)	40 (22.2)	119 (66.1)	19 (10.6)	2 (1.1)	0 (0)
Crisis teams (*n* = 180)	30 (16.6)	92 (51.1)	47 (26.1)	6 (3.3)	5 (2.78)
Third sector-commissioned providers (e.g. dementia advisors) (*n* = 180)	22 (12.2)	119 (66.1)	20 (11.1)	5 (2.8)	14 (7.78)
General adult CMHTs (*n* = 181)	19 (10.5)	91 (50.3)	57 (31.5)	6 (3.3)	8 (4.4)
Care homes (*n* = 179)	17 (9.5)	123 (68.7)	33 (18.4)	1 (0.6)	5 (2.79)
Social services (*n* = 180)	13 (7.22)	81 (45)	74 (41.1)	12 (6.7)	0 (0)
General hospital in-patient services (*n* = 179)	10 (5.59)	76 (42.5)	63 (35.2)	11 (6.2)	19 (10.6)
Primary care (*n* = 180)	9 (5)	100 (55.6)	66 (36.7)	4 (2.2)	1 (0.56)
General hospital out-patient services (*n* = 179)	4 (2.23)	41 (22.9)	41 (22.9)	13 (7.3)	80 (44.7)

CMHT, community mental health team.

a. *n* refers to the number of services who provided a response to the question.

The services with whom relationships were most frequently rated as poor or requiring improvement were social services (86, 47.8%), general hospital in-patient (74, 41.4%) and out-patient (54, 30.2%) services, and primary care (54, 30.2%). Across services, issues of communication, responsiveness, lack of integration and inefficiency of services due to being under resourced were identified as key areas of improvement.

### Views about service successes, concerns and expectations

We integrated responses to these questions and present them using chapter headings from the UK Government’s 10-Year Health Plan for England as a framework (Supplementary Tables 1–3).^23^

#### From hospital to community

Commitment to delivering integrated care featured prominently in responses; where this worked well, ‘patient has access to more [services]’. Unintegrated working was a major concern. One respondent described how service pressures adversely affected inter-service relationships:

‘Reduction in social care provision, reduction in bed availability, pressures in primary care meaning that there is potential for relationships to be worse in primary and secondary care – everyone is so stressed and pressured – always push back on who should be doing what…the bottom line is that all of this increased stress on patients and families – it is harder now to be older with mental health problems than it was 5 years ago’.

Positive service developments included integration with social care, a local frailty hub, acute medicine, primary care and other mental health services. Many respondents were proud to provide a timely service and concerned about the impact of resource limitations on waiting times. Resource concerns extended to social care and third-sector partners:

‘Lack of accessibility to social care; continue to hold patients for longer than need which increases pressure on OPCMHT as they’re waiting for social care to be put in place’.

Many respondents noted increasing complexity of caseloads, in part because of a shift from hospital to community care, which they expected to increase in the next 5 years:

‘Might cut more in-patient beds due to changes in mental health legislation, so services will be busier as people will need to be at home more’.

There was also an expectation that fewer complex cases would be seen in primary care:

‘Will be seeing more complex patients… patients with a less higher need will be seen in primary care’.

In line with survey responses above, respondents noted that services received more referrals from people with complex emotional needs, personality disorders and with drug and alcohol concerns, including comorbidity with dementia. One service described how ‘areas of the service are being developed for complex emotional needs ([through more] funding for complex emotional needs/personality disorders) for older adults’.

Some respondents described ‘intervention-based’ or ‘episodic care’, in which clients were accepted for discrete courses of treatment such as psychological therapies, as ‘good flow’. For others, it was perceived as a barrier to delivering a holistic, person-centred service:

‘Episodic care model (discharging people quickly) – some staff and patients find that quite difficult and uncomfortable’.

Eleven services described initiatives to reduce hospital admissions and enable timely discharges, including access to home treatment teams and other innovations, for example:

‘We are looking to get a 4-bedded unit within this team, so that patients do not have to go to hospital’.

Other initiatives to increase accessibility of services included OPCMHT clinics in general practices, and in one service, plans to introduce self-referral.

#### From analogue to digital

Several respondents spoke positively about the adaptations they had made to include online and telephone consultations alongside face-to-face options. Some expressed concern that services that were now remote by default such as NHS Talking Therapies and primary care could exclude people who cannot access digital technologies. In the next 5 years, they anticipated greater use of wearable technologies, electronic prescribing (which has been present in primary care for some time) and artificial intelligence for communication (e.g. clinic letters).

#### From sickness to prevention

One respondent saw current care for people with mild cognitive impairment (MCI) as a missed opportunity for prevention:

‘People with MCI may not receive the same opportunities of treatment (no clear pathway, caught up in DDR [dementia diagnosis rate], people with MCI tend to get missed and there is less focus on prevention)’.

Others described initiatives to embed services in frailty and anticipatory care hubs, which could potentially detect issues earlier, enabling secondary or tertiary prevention.

#### A new transparency of quality of care

Respondents were proud of meeting key performance indicators (used to measure performance and quality of the service) and receiving positive client feedback, with one service adding that ‘it feels like [the service] do make a difference’. However, resource limitations were highlighted as a major challenge to care quality. One respondent commented that: ‘[resource constraints] cause services to be pressured and they try to keep patients out, [as] over capacity.’ This left another respondent ‘worried about how to safely provide care and support for people if we keep being stripped of resources and funding’.

Another was concerned about value for money that might be spent on disease-modifying therapies (DMTs) for people with Alzheimer’s disease:

‘Services need to be careful in making sure that it gives the benefit to the cost. DMTs tend to be more accessible to the top percentile but not sure if it necessarily does any good’.

Some were concerned that a lack of specialist expertise, including in service leaders, will reduce quality of care. One respondent expressed concern at a lack of productivity from staff and professional curiosity, perhaps describing burnout.

#### An NHS workforce, fit for the future

Respondents were proud of staff retention, values, teamwork, skills and newly created workforce roles, such as nurse medical practitioner. Staff stress and high turnover were major concerns. In the next 5 years, they anticipated new roles, with a focus on therapy delivery and primary care liaison. Services anticipated the need to upskill staff in the areas of physical health monitoring, therapy delivery and supporting evolving client needs – including managing alcohol misuse and dementia. Fourteen respondents commented on the significant change in skills mix needed if DMTs for Alzheimer’s disease were introduced.

## Discussion

This is the first national survey of older people’s community mental health services in England for over a decade. Collectively, these teams receive over 120 000 referrals a year. Although many were proud of providing timely, integrated services for older people with severe mental health symptoms, including those with dementia, a quarter of respondents referred to waiting times of over a month, exceeding recommended limits.^26^ Clients in the North-East and Yorkshire regions were four times as likely to wait this long as compared with those living in London and North-West regions. Half of services reported difficulty accessing in-patient beds for people with dementia, and nine in ten reported that service-related factors delayed hospital discharges. These challenges were greater in more rural regions, perhaps relating to acknowledged difficulties in the provision of domiciliary care in these areas.

CMHTs have diverse functions, as evidenced by varying service structures and process. In a *post hoc* analysis, we found that services with separate memory assessment teams, and services that were distinct to older adults, were more likely to see clients within a month for initial assessment (Supplementary Table 5). Further research could expand on the effectiveness of these service organisation structures.

Almost half the respondents described relationships with social services that were poor or required improvement, and over a quarter reported similarly problematic relationships with secondary and primary healthcare services. Despite this, half of services reported providing higher quality of care to people with dementia than 5 years ago, even with increasing caseload complexity. Some respondents perceived tensions between episodic, intervention-led care models (where clients were engaged in discrete interventions then discharged) and person-centred care.

Compared with the 2008 survey, we identified fewer, smaller teams; this is to be expected because of the creation of memory services since this time. In 2008, teams surveyed had on average 18.1 team members and received 36 referrals per month (24 referrals per team member per year). Although methodological differences preclude direct comparisons, this is indicative, in common with the responses to our survey by participants, of increased workloads. Relative to 2008, we found a more multidisciplinary skill mix, with slightly fewer doctors and more occupational therapists and clinical psychologists, as well as a marked increase in the proportion of local care homes receiving regular input from mental health services.^22^ Although the Community Mental Health Framework for Adults and Older Adults recommends social workers among the core team,^28^ most services did not have this provision, instead liaising with social services externally.

### Clinical implications

The expertise of staff working in OPCMHTs will be vital to delivering integrated, neighbourhood health services, so the consistent commitment to closer collaboration with other services is very positive. Closer working with the voluntary sector, and increased patient involvement in service planning and care is anticipated. However, the pervasive low level of resources underpinning evidence-based care in current systems is concerning. There is considerable diversity between countries in terms of how services for mental health of older people are configured. Globally, dedicated specialist mental health services for older people are not common, although a 2003 Australian survey had similar findings relating to variability in structure, resource limitations and workforce training.^34^

Respondents reported recent and anticipated shifts in workforce skill mix to support greater therapy delivery. Appropriate treatment for depression and anxiety can help prevent or delay dementia,^5^ and effectively treat people with dementia, with large effect sizes;^35^ but two in ten services did not offer individual therapy to people with dementia, and three in ten did not offer individual carer support, which is cost-effective.^36^ This echoes findings from our recent survey of memory services that a third do not offer cognitive stimulation therapy, despite National Institute for Health and Care Excellence guidance and evidence of cost-effectiveness.^15^ Greater allocated resource for evidence-based therapies could support shifts toward more preventive and higher quality of care, especially since waiting times for clinical psychology within OPCMHTs were often long. This will particularly affect people with dementia, as many NHS Talking Therapies services feel ill-equipped to work with people with dementia,^35^ so cases are frequently referred to OPCMHTs.

The lack of therapies offered to people with mild memory concerns without dementia was noted by one respondent as a missed prevention opportunity. Any plans to resource DMTs for people with dementia, anticipated by many participants, must consider these opportunity costs to the delivery of psychological therapies to help prevent and treat dementia.

Although respondents showed a commitment to care integration, they also identified skill gaps around physical care needs and therapy delivery. Proposals in the 10-Year Health Plan for England around personalised, lifelong learning for the health and care workforce could, if well-implemented, address these skill gaps. DeNPRU-QM has called for mandatory, evidence-based training across health and social care, investing in experiential learning and cross disciplinary training.^37^ Although shifts to more episodic care models may be perceived by some to threaten person-centred care, skills of empathy and person-centredness can be learned, and in integrated models, wider teams can provide safety nets for vulnerable clients.^13^

### Limitations

Our survey has important limitations. There may be responder bias, potentially favouring services under less strain, but the high response rate is encouraging. We did not independently verify data, which may have been subject to desirability bias. Although it was unclear how to compare this, the trend shows that referrals have increased substantially in the past 5 years in most services.

Many services were in the midst of mergers and reorganisations at the time of the survey, and we identified fewer services than a previous survey. This probably reflects the mergers and therefore a true reduction in the number of teams, although it is also possible that we failed to identify some services.^21^ To mitigate this potential sampling bias, we iteratively updated our sampling frame as respondents informed us of other services in the area or within respective Trusts. We compared FTE between regions, but this did not account for skill mix. We identified difficulties in recruiting certain positions (most frequent response being senior clinicians or nurses), so it is possible that services with higher FTE may nonetheless have low FTE of decision makers and specific skills needed to provide a good-quality service. We recorded most recent CQC rating as an external quality metric, but most ratings were from 2020, and therefore did not provide a contemporary metric of care quality. Where services were combined with memory services, we did not elicit further detail of how allocation of clients and pathways of care were configured. Case studies illustrating effective structures may be helpful for service commissioners.

In conclusion, OPCMHTs can be integral to the success of future neighbourhood health services and shifts toward a greater focus on prevention. Addressing workforce retention and resource limitations (e.g. in social care and upskilling in therapeutic and physical health skills) could help realise their potential contribution to future dementia prevention and care, and support a shift from hospital to community-based care.

## Supporting information

Muralidhar et al. supplementary material Muralidhar et al. supplementary material

## Data Availability

The data that support the findings of this study are available from the corresponding author, M.M., upon reasonable request.

## Supplementary material

The supplementary material is available online at https://doi.org/10.1192/bjo.2025.10958
