# Supplementary material for: National survey of older people’s community mental health teams in England
Source: BJPsych Open. 2026 Jan 26;12(1):e50. doi: 10.1192/bjo.2025.10958 (PMC12835709; doi:10.1192/bjo.2025.10958)
Supplement: Muralidhar et al. supplementary material [file S2056472425109587sup001.docx]

**Supplementary material**

**Table S1: Qualitative coding summary for ‘What aspects of your service in the past 5 years are you most proud of?’ (n=164; reporting on 181 services)**

| **Code** | **N** | **Examples of items coded** |
| --- | --- | --- |
| **Hospital to community care: the neighbourhood health service as patient-centric, integrated care** | | |
| Timely service | 62 | “Reduced waiting time for assessment from around 36 days down to around 18 days on average over the last year. More consistently hitting targets in terms of 4 week wait target”  “I feel proud that we are an accessible service and provide interventions in a really timely manner. |
| Developing new services | 28 | “Development of dementia home treatment team”  “Areas of the service are being developed for complex emotional needs (CEN) (funding for CEN/personality disorders) for older adults.” |
| Integrated working | 22 | **“**Improved engagement with primary care and social services. Getting a lot better at joint working.”  “Integrated working so that patient has access to more (e.g., memory service also had access to care home liaison etc).”  “Comprehensive range of services provided under SAMH (memory, CMHT, HTT, recovery service, care home liaison, diagnosis to end of life pathway - all bases are covered)”  “Interface work with inpatient areas, lots of value on relationships between teams”  “How the team have transitioned from just dementia specific to people with mental health conditions, they have transitioned really well and I feel we provide a good service to those patients as well.” |
| Intervention-based service | 11 | “Good flow, don't keep people on unnecessarily, assess and then discharge back to the GPs and all the agencies that might support them in their journey.” |
| Inclusive, person-centred holistic care | 16 | “Continue to provide a good largely holistic patient care focused on the needs of the individual rather than templates.”  very holistic assessments and treatments that involves the family members and friends (if appropriate)”  “Tailor interventions to support patient needs (person centred care)”  Very person centred and advocacy for patients is great. |
| **Sickness to prevention** | | |
| Inclusivity | 3 | “The work we have done to identify patients under the case load who have not been seen has brought forward many patients to be signposted and receive the care that they previously weren't. Not letting people fly under the radar.”  “Service has responded to demands of local demographic, trying to tailor service provision to demographic (cultural diversity, DEI), more engaging with other services e.g. AgeUK. |
| **Analogue to digital: a single patient record - to enable more co-ordinated, personalised and predictive care; allow patients to leave feedback on care received** | | |
| Adapting approaches | 2 | “Learnt to adapt and deliver care in different ways (face to face, telephone, online etc - whereas it was all face to face previously) - gotten better at using technology and being more creative and flexible in meeting patient needs.” |
| **NHS workforce fit for the future; acquiring new skills, supported by personal development plans** | | |
| Staff retention | 16 | “Very good retention of staff, service is seen as a positive place to work” |
| Staff values | 23 | “Kind and compassionate, skilled group of practitioners. Proud of their ability to rise to new challenges and integrate changes as required.” |
| Teamwork | 4 | Team work extremely well in responding to crises and high risk |
| Adapting to limited resources | 2 | “stepping up to challenge of meeting needs of increased referrals despite the lack of resource.” |
| Staff recruitment | 5 | “Recently had a new manager and team was previously short staffed - now fully staffed” |
| Staff skills | 10 | “I am proud of the training that the trust offers to the staff. It gives a lot of skills and knowledge.”  Knowledge and expertise in working with older adults is great.  encouraging staff to be aware of latest practices/updates, staff commitment and drive” |
| Staff morale | 13 | “Sticking together as a team, supportive of each other.”  “Morale has improved from ensuring regular supervision for all and reflective practice group for all staff.” |
| New workforce roles | 1 | “Creation of nurse medical practitioner post has created more time for psychiatrists and a pathway for if medications prescribed |
| **New transparency of quality of care; informed by patient experience** | | |
| Positive patient feedback | 3 | Consistency of patient and carer positive feedback on the dementia service offered. “We maintain retention of staff and we get very positive feedback from service users.” |
| Meeting KPIs/Quality improvement | 9 | “Meeting targets, lowering wait times, service improvements (e.g post-diagnostic group)”  “Good with all the KPIs of services, and quality improvement projects”  “Quality improvement project currently underway which is attached to the LEC project on engagement and collaboration/coproduction.” |
| Maintaining service quality | 26 | “Continuing to provide consistently good service despite financial pressure and cutbacks.”  “Didn't stop running during Covid and were able to deliver services to people. Have gone through difficult times with staffing but have been able to retain quality of care and MSNAP accreditation” |

**Table S2: Qualitative coding summary for ‘What changes in the service that clients receive in the last 5 years concerns you the most?’ (n=164; reporting on 181 services)**

| **Code** | **N** | **Examples of items coded** |
| --- | --- | --- |
| **Hospital to community: the neighbourhood health service as patient-centric, integrated care** | | |
| Increased wait times for services | 22 | “Staffing challenges that have led to waiting lists, that are really hard to catch up with, waiting times have increased”  “Long waiting list for psychology, especially patients waiting for neuropsychology assessment (deteriorating while waiting for assessment)” |
| Demand exceeding resources | 48 | “Due to financial issues, we are unable to use other contract agency or bank staff, even if the needs rise.”  “Staffing levels don't reflect the number of referrals we receive” |
| Limited social service support | 16 | “Becoming harder for the client group to access social support services especially home care”  “day centres have closed down that used to help people live independently for longer.”  “Lack of accessibility to social care; continue to hold patients for longer than need which increases pressure on OPCMHT as they're waiting for social care to be put in place.”  “Lack of funding of the 3rd sector which directly impacts patients and carers” |
| Fewer inpatient beds | 13 | “Taking longer to get patients admitted into hospital as dementia beds have been reduced. Resulting in delayed admissions.”  “Older adults dementia in patients ward has closed so the care provided to ensure that people are safe at home has had to adapt.” |
| Increased complexity | 12 | “More people with complex emotional needs, starting to look at training for staff to work with these patients”  “Receiving more transfer of care for older adults coming into the service with more complex presentation and higher risk medication.” |
| Barriers to discharge | 7 | “Some of our GPs will not prescribe anti-dementia medication so we then take these cases on and they are then on our books for way too long.” |
| Disintegration of services | 11 | “Segregation between health and social care is concerning”  “Getting things done for the better of the patient has slowly reduced; gap between physical and mental health services, mostly due to funding issues. “people end up bouncing back and forward between team which leads to opportunity for delays.”  “Reduction in social care provision, reduction in bed availability, pressures in primary care meaning that there is potential for relationships to be worse in primary and secondary care - everyone is so stressed and pressured - always push back on who should be doing what…the bottom line is that all of this increased stress on patients and families - it is harder now to be older with mental health problems than it was 5 years ago.”  “Explosion of specialist care homes in the geographical area with little consideration in the planning of the healthcare provision.” |
| Intervention-led services | 12 | “Episodic care model (discharging people quickly) - some staff and patients find that quite difficult and uncomfortable.”  “Previously offered a gold standard (assessment, diagnosis, end of life pathway) - no longer see patients all the way through due to increased volume of referrals. Now only diagnose, treat, signpost and discharge”  “More intervention-led service but there are some patient groups who may benefit from regular contact rather than a specific intervention, which the service isn't always able to provide.” |
| Increased documentation/ non-clinical activity | 8 | “Increased documentation and legislative requirements from nurses - takes away from patient contact time”  “Expectation from higher management that all staff will be involved in managing complaints and rapid learning reviews which takes away time from seeing patients and attending to caseloads (takes more time).” |
| **Analogue to digital: a single patient record - to enable more co-ordinated, personalised and predictive care; allow patients to leave feedback on care received** | | |
| Adapting to new approaches | 6 | “Need to get used to the new system. Social care records system for commissioning care can be difficult to use in comparison to paper form and so are now running 2 systems, both mental health records and social care records system.” |
| Digital exclusion | 2 | “IAPT services used to provide GP based appointments but have all gone online since covid. Has not gone back since covid so very limited face to face provision and requires people to travel a long way. Has put a lot more pressure on the OPCMHT, feels like they hare having to plug the gaps and help people who would benefit from standard CBT.” |
| **Sickness to prevention** | | |
| Missed opportunities for secondary prevention | 1 | “People with MCI may not receive the same opportunities of treatment (no clear pathway, caught up in DDR (Dementia Diagnosis Rate), people with MCI tend to get missed and there is less focus on prevention).” |
| Missed opportunities for tertiary prevention | 8 | “The limitations to therapeutic approaches, specifically with the services limited psychology resources. Having preventative approaches rather than using a medication model.”  “The delay in diagnosing dementia is adversely impacting patients, carers, and services. Waiting for a diagnosis means that people cannot access the services and support they need. this can escalate and become more risky.”  “Wait for packages of care and social care crises that could be avoided which affects patients with functional mental health problems and/or living with dementia more frequently.” |
| **NHS workforce fit for the future; acquiring new skills, supported by personal development plans** | | |
| Staff turnover/ recruitment issues | 9 | “Staffing is under strict caps and controls because of the financial service. When people leave haven't been able to recruit back in.”  “Staffing is quite low at the moment, issues finding and recruiting staff”  “Time taken to recruit new staff has increased” |
| Staff stress | 2 | “People are becoming very stressed due to the constraints happening within healthcare (e.g., even if you have a member of staff leaving, they will not be replaced). This causes people to break down and have lots of sickness.” |
| Lack of training/ skills specific to older people | 6 | “[not] Following evidence-based pathways, sitting on caseloads without clear interventions, ongoing issue rather than change”  “Quite chaotic since merger. Lack of staff with experience (especially for older adults so relying on agencies but this has been an issue), recruitment is an issue. Lack of productivity from staff and professional curiosity.” |
| **New transparency of quality of care; informed by patient experience** | | |
| Lack of expertise within leadership/ management | 5 | “Lack of leadership with any of the senior leaders having expertise in older people's mental health.”  “Changes have been destabilising for management as communication is not clear between managerial levels.” |

**Table S3: Qualitative coding summary for ‘What changes do you anticipate in the next 5 years?’ (n=163; reporting on 179 services)**

| **Code** | **N** | **Examples of items coded** |
| --- | --- | --- |
| **Hospital to community: the neighbourhood health service as patient-centric, integrated care** | | |
| Increased demand and same/fewer resources | 35 | “Trust is in difficult financial situation and posts are freezing, which may lead to increased waiting times and more pressure on beds.”  “Will continue to be a challenge because every service is under the same pressures of increased workload without increased resource, and that creates services becoming protective of their criteria, which negatively impacts working relationships with other services”  “Concerned about the gradual diminution of council and 3rd sector support for people with dementia e.g., closing of day centres and the pressure this may cause for patients, carers and healthcare services.”  “Will continue to see increase in big nursing homes being built, that has an impact on team resources that we can provide.” |
| More integration | 98 | “Will have to become increasingly linked in with social care and colleagues in acute medicine as people are living longer and have more complex needs”  “In [area] we are looking to have an integrated team and have a social worker within our team”  “Beginning to make more links with local frailty hub and will support them make dementia diagnosis if they need to and also look at how their pathways flow into the service”  “Transformation will change the way they service works with other services. Better access to home treatment teams and crisis service.”  “closer relationship with primary care and are able to reduce barriers to referrals and provide prompt advice and intervention.” |
| Increased complexity | 13 | “Might cut more inpatient beds due to changes in mental health legislation, so services will be busier as people will need to be at home more.”  “Will be seeing more complex patients… patients with a less higher need will be seen in primary care”  “Higher thresholds, working with more acutely unwell clients” |
| Creating specialist dementia services | 12 | “Developing standalone memory assessment service which will allow more capacity to really engage with stakeholders.”  “Inpatient beds are going to be separated into dementia specific wards and mental health wards which will hopefully mean improved specialised care “ |
| Reduced specialisation focus | 3 | “Concerns around becoming one team as older adult specialty is going to get diluted due to this.” |
| Improving access to services | 9 | “considering allowing patients to self-refer if they are already in our system. This also allows patients and their families to be more autonomous.”  “Work in progress to establish clinics outside communities (e.g., seeing clients at GP surgeries so that people don't have to travel too far).” |
| New services to avoid admissions | 11 | “We are looking to get a 4-bedded unit within this team, so that patients do not have to go to hospital.”  “We are trying to develop a better 7-day-a-week-service to try and prevent admissions.” |
| Analogue to digital: a single patient record - to enable more co-ordinated, personalised and predictive care; allow patients to leave feedback on care received | | |
| Technological developments | 9 | “Potential to provide better communication with the rise of AI resources”  “Think we will be doing electronic prescribing with less reliance on GPs for prescribing.”  “Implementation of AI and wearable technology which will improve patient care.” |
| More online consultations | 1 | “Will gradually do more online, increasing IT literacy in older people |
| Sickness to prevention | | |
| Secondary prevention | 12 | “More embedded in frailty and anticipatory care hubs.” |
| NHS workforce fit for the future; acquiring new skills, supported by personal development plans | | |
| Different skills mix | 6 | “Looking at how the workforce will look in the future, if the service needs more therapy based staff and less nursing”  “New therapies are coming that will change the way of working” “Development of primary care liaison role to support GPs.” |
| Changes if DMTs introduced | 14 | Development of effective treatments for (Alzheimer's) dementia … complicated to deliver and require specific training needs.” |
| Upskilling, physical health | 3 | “GPs have now been decommissioned to do ECGs for patients under the CMHT, so there will be increase in the CMHT needing to provide the physical health support.”  “We are probably going to have to train our staff up to do more physical health monitoring and neuroimaging.” |
| Recruitment challenges | 10 | “Recruitment of psychiatrists a big challenge, might result in more of the service being nurse-led with less doctors.” |
| Training/upskilling mental health | 6 | “We are doing a lot of work in the functional pathway so that staff can develop their skills and learn how to deliver various therapies.”  “We are going to train in Open Dialog - a model for communicating with people with mental health difficulties.”  “trauma informed care: there has been more training around this recently… potentially looking to introduce cognitive stimulation therapy which is in the NICE guidelines but not currently offered by the service”  “more referrals regarding alcohol, … So feel as though they have a lot of learning to do to meet the needs of a rapidly changing older population.” |
| Staff retention and increased staffing | 7 | “Hope for more consistency in staffing and staff retention, which leads to stability in team dynamic and more experienced staffing group”  “Increasing the workforce to meet the demand.” |
| New transparency of quality of care; informed by patient experience | | |
| Increased service user involvement | 5 | “Already seeing increased involvement with service users and carers (including in decision making regarding services); employed peer support workers”  “Working more towards collaborative working the patients, co-creation is a big push within the Trust.” |
| Intervention-based care | 2 | “looking more towards intervention-based care” |
| Cost-effective care |  | “A DMT will be rolled out and so services need to be careful in making sure that it gives the benefit to the cost. DMTs tend to be more accessible to the top percentile but not sure if it necessarily does any good.” |
| Service development/ restructuring | 25 | “Further development of care pathways within the service”  “There is currently a pathway review going on”  “Further internal restructuring which may affect ways of working.” |
| Increased pressure reducing service quality | 4 | “Highly likely to have increase in comorbidities that require co working with other providers but this causes services to be pressured and they try to keep patients out, over capacity.”  “Worried about how to safely provide care and support for people if they keep being stripped of resources and funding.” |
| Streamlining processes | 4 | “Discussions happening about changes in structure, no wrong door service, to improve patients journey and prevent rereferrals, make us all one team. Prevents patient having to repeat their story to multiple professionals, more streamlined approach to care.” |

**S4: Full survey of questions asked to participants**

**Service structure**

1. Do you have:
2. a service dedicated to assessing and treating mental illness in older people (65+) and which does not include the main service for assessment and diagnoses of dementia?

Yes

No

1. a service dedicated to assessing and treating mental illness in older people (65+) and which also assesses and diagnoses dementia?

Yes

No

1. a service dedicated to assessing and treating mental illness in one team for adults of all ages?

Yes

No

If yes (to iii), does this team also assess and diagnose dementia

Yes

No

1. a separate service that focuses on the assessment and diagnosis of dementia (a memory service)?

Yes

No

No –services are provided in another way (specify ________________________)

1. What is your staffing mix?

____ FTE Psychiatrists

____ FTE Mental Health Nurses

____ FTE Consultant Nurses

____ FTE Clinical Psychologists

____ FTE Occupational Therapists

____ FTE Support Workers

____ FTE Social Workers

____ FTE Band 4 Therapists

____ FTE Advanced Practitioners

____ FTE Administrative Staff

____ Other

If other, please specify: ______________________

2b. Are there any specialities you are currently having issues recruiting?

**Please note, all following questions in the survey refer to older patients (65+) only.**

1. Does your team provide home treatment/crisis services?

Yes

No, a separate team provides this

No, no such team exists

Comments:

1. Does your team provide a care home liaison facility (staff whose role is to provide psychiatric services to care homes)?

Yes

No, a separate team provides this

No, no such team exists

Comments:

1. Approximately how many people were referred to your service in the last year?

_________

5b. How has this referral rate changed compared to 5 years ago?

Increased

Decreased

Stayed the same

5c. If it has increased or decreased, what do you think may have influenced this?

1. Does your service allow self-referrals?

Yes

No

(if yes) 6b. Approximately what proportion of your referrals in the last year have been self-referrals?

___________

1. Where does your service see new clients with dementia with mental health issues living in the community?

% In their homes

% In the clinic

% Online/telephone

% Other

If other, please specify: ___________________________

**Patient characteristics and care**

1. After referral, what is the average waiting time for patients to receive an initial assessment?

1 week

2 weeks

1 month

Over 1 month

Not sure

Comments:

1. On average, approximately how long after initial assessment do patients wait for these services? How has this changed in the last 5 years?

[Tick N/A if service not available]

| **Service** | **N/A** | **Wait for service after initial assessment** | **Over last 5 years: Increased/decreased/stayed the same?** |
| --- | --- | --- | --- |
| Allocation to a care coordinator |  |  |  |
| Appointment with a clinical psychologist |  |  |  |
| OT assessment |  |  |  |
| Physiotherapy assessment |  |  |  |
| Appointment with a speech and language therapist |  |  |  |
| Appointment with dietician |  |  |  |
| Medical outpatient review appointment |  |  |  |
| Social services assessment |  |  |  |
| Other, specify:  ______________ |  |  |  |

1. Has your service seen a change in the proportion of clients presenting with the following conditions?

| **Conditions** | **Y/N** | **Increased / decreased** |
| --- | --- | --- |
| Dementia |  |  |
| Harmful use of alcohol |  |  |
| Harmful use of illicit drugs |  |  |
| Personality disorder |  |  |
| Late onset psychosis |  |  |
| Anxiety |  |  |
| Depression |  |  |
| Autism |  |  |
| Cognitive impairment following pre-existing psychosis diagnosis |  |  |

Comments:

1. Is your service experiencing an ongoing backlog since the COVID-19 pandemic (e.g., waiting times that have not recovered from pre-pandemic levels)?

Yes

No

Comments:

**Work with other services**

1. Beyond co-working for specific clients, does your service have any regular liaison with the following services? Please provide details.

| **Service** | **Y/N** | **How often** | **Details** |
| --- | --- | --- | --- |
| Primary care |  |  |  |
| Domiciliary services |  |  |  |
| General hospital services |  |  |  |
| Care homes |  |  |  |
| Day care services |  |  |  |
| Organisations for minoritised groups |  |  |  |
| Other |  |  |  |

1. How do you find the interface between your service and the following services?

|  | **N/A** | **Poor** | **Requires improvement** | **Good** | **Excellent** |
| --- | --- | --- | --- | --- | --- |
| General adult CMHTs |  |  |  |  |  |
| Memory services |  |  |  |  |  |
| Social services |  |  |  |  |  |
| Crisis teams |  |  |  |  |  |
| Care homes |  |  |  |  |  |
| Third sector-commissioned providers (e.g., dementia advisors) |  |  |  |  |  |
| Primary care |  |  |  |  |  |
| Mental health wards |  |  |  |  |  |
| General hospital inpatient services |  |  |  |  |  |
| General hospital outpatient services |  |  |  |  |  |

Comments / what could be improved?

1. When they are required, do you have difficulty accessing in-patient beds for people with dementia with mental health problems?

Yes

No

Comments

1. Are there service barriers that prevent timely discharge of people with dementia and mental health problems into the community?

Yes

No

Comments:

**Further support and engagement**

1. Which of the following therapies are typically offered to patients with dementia with depression and/or anxiety?

Individual session with carers

Individual therapy for people with dementia

Groups for people with dementia

Carer groups

16b. Please provide details.

1. Do you offer any specific support or services for people with dementia from the below communities? If so, please provide details about the support you provide.

|  | **Y/N** | **Type of support** |
| --- | --- | --- |
| Ethnic minority communities |  |  |
| LGBTQIA+ community |  |  |
| Veterans |  |  |
| Other_________________ |  |  |
| Other_________________ |  |  |

1. Are patients with dementia routinely offered opportunities to take part in research?

Yes

No

18b. How has engagement of your patients/service with research changed in the last 5 years?

No change

Lower engagement

Higher engagement

Unsure

1. What aspects of your service in the past 5 years are you most proud of?

______________________________________________________________________________________________________________________________________________________

1. What changes in the service clients receive in the past 5 years concerns you the most?

______________________________________________________________________________________________________________________________________________________

1. In the last 5 years, how would you describe any change to the quality of care for people with dementia you provide:

No change

Lower quality

Higher quality

Not sure

Comments:

22. What changes do you anticipate in your service in the next 5 years?

22a. To how you work with other services?

22b. Anything else?

**S5: Post hoc analysis of wait times for initial assessment based on service structure**

|  | **Wait times for initial assessment within 1 month** |
| --- | --- |
| **Separate memory service (N = 155)** | 117 (75.5%) |
| **Older people’s service only (N = 145)** | 108 (74.5%) |
| **Combined memory service and CMHT (N = 27)** | 13 (48.1%) |
| **All ages service (N = 37)** | 22 (59.5%) |
| **All ages CMHT that also diagnoses dementia (N = 31)** | 18 (58.1%) |
